# A New Operation for the Cure of Varicocele

**Published:** 1843-03-04

**Authors:** J. Pancoast

**Affiliations:** Professor of Anatomy in Jefferson Medical College of Philadelphia, Lecturer on Clinical Surgery, &c.; Philadelphia


### A NEW OPERATION FOR THE CURE OF VARICOCELE.

BY J. PANCOAST, M. D.,

Professor of Anatomy in Jefferson Medical College of Philadelphia, Lecturer on Clinical Surgery, &c.

*To Dr. Clymer:—*

MY DEAR SIR,—I send, at your request, the following account of a method which I have twice employed with success for the cure of the varicose enlargement of the veins of the spermatic cord and scrotum, known under the names of cirsocèle and varicocele.

Previous to the operation, the patient is to be directed to walk about for an hour or two with the scrotum unsupported, so as to cause an accumulation of blood in the enlarged veins. He is to be seated on the side of his bed, with the legs separated. The thumb and forefinger of the left hand are then to be pressed in, so as to lift up the enlarged veins, and thus separate them from the vas deferens. This duct is readily distinguished by its hard and wiry feel, and is to be pressed off with the nail of the left forefinger towards the os pubis. A long, round lancet-pointed needle, curved near the point, like that of the sail-makers', threaded with a piece of fine but strong hempen twine passed double through the eye, is then to be carried between the bundle of veins and the vas deferens; entering it on the side of the thumb, and bringing out the point against the pulpy portion of the finger. The loop of the double ligature is to be detached from the needle; the ligature being left in the track of the wound. The needle, without being threaded, is again to be entered through the same orifice of the skin as before, but carried this time between the skin of the scrotum and the veins of the cord, and its point brought out through the other puncture made in the skin on the side next the pubis. To facilitate this step, the skin should be lightly raised up from above the veins with the thumb and finger. If there is any enlargement of the subcutaneous veins of the *front part of the scrotum*, as there was in one of my cases, I carry the point of the needle so as to scrape the under surface of the skin, and get it in front of these veins. The needle is now to be left in the wound. I manage to have the place of entry of the needle lower than its point of exit; so that the point of the instrument, which should be pushed well through, may lay undisturbed, without pressing over the root of the penis. The course of the instrument across the cord will be, therefore, rather diagonal than transverse. The loop of the ligature (which lies next the pubis) is now to be thrown over the point of the needle. Traction is next to be made upon the other side, upon the loose ends of the ligature, so as to draw the loop along the needle, through the orifice in the skin. One tail of the ligature is now to be drawn out for four inches, so as to shift the portion of the thread, forming the loop over the needle, for fear that this might have been cut by the point or edge of the needle, so as to break when subsequently knotted. The loose ends of the ligature are then to be drawn tight, and tied with a single knot over the shank of the needle; this is to be drawn as tight as possibie, so as to completely strangulate the veins of the cord, which will be thus enclosed by the double ligature on its back part, and the needle in front. To make the strangulation more effectual, the two ends of the loop thus formed over the needle may be slid towards each other, by pressure through the skin, and the knot again tightened. Severe pain follows this step, which gradually diminishes, and at the end of half an hour ceases almost entirely. To be able to tighten the ligature again at the end of two or three days, when it will be found loosened by having partially cut through the compressed mass of veins, I slide an oblong piece of sole leather, pierced in the centre and notched at the ends, over the heel of the needle, and make a firm double bow knot of the ligature above it. The point of the needle is to be sheathed in a small cork, and a compress placed below it to prevent its worrying the skin. A piece of thick tape is to be passed through the eye of the needle and knotted, in order to prevent it when it has become loosened by suppuration, from being pressed through the hole in the leather, by the movements of the thigh, so as to loosen the loop. The scrotum is to be slightly supported by a couple of silk handkerchiefs, folded, and placed below it. No dressing is required. If neuralgic pains arise, they are to be soothed by hot fomentations, and the administration of anodynes. I untie the ligature over the leather every third day for three successive periods, tightening it as much as possible each time. On the eleventh I remove the needle; the loop, which is then left detached, and has become very small from the successive tightenings, is at the same time withdrawn. Above the place of the ligature, the condition of the cord at this time appears perfectly natural; below it there is a hardened mass of the size of a walnut, formed by the effusion of lymph, between, and in all probability in the cavities of the veins so as to obliterate them entirely. The pain attending this process of cure is but trifling, except at the periods when the loop is tightened. There is no injury done to the integument, such as to leave a scar after the cure is completed, for the needle, if introduced in the manner I have mentioned, lays so completely at rest, as to cut but very slightly at the places of puncture, and as it makes no pressure in the downward direction, cannot by any possibility impair the integrity of the vas deferens. After the withdrawal of the needle, a light poultice may be laid for a few days over the part, to promote suppuration from the points of puncture, and to facilitate the resolution of the tumour left, a result which is quickly effected.

The advantages of this method of operation will, I think, be found sufficient to recommend it to the notice of practitioners. The plan of cure recommended by Sir A. Cooper, which involves the excision of a part of the scrotum, is severe, dangerous, and inefficacious. The methods of Breschet and Ricord are complicated by the use of a cumbersome apparatus. That of Reynaud is attended with a division of the integuments, which leaves, like the three former, a permanent cicatrix, and the modification of this, as suggested by M. Vidal, appears by no means free from objections.

The two latter I have, however, successfully employed, but the treatment of the cases have been necessarily longer, and attended by a much greater amount of suffering. In one instance, after treatment by Ricord's method, an enlargement of the veins subsequently reappeared.

By the method which I have proposed, it is possible at any moment, in case the strangulation of the veins and nerves of the cord should give rise to obstinate neuralgic pains or retention of urine, to relieve the patient by slackening temporarily the ligature, and to shorten the period of treatment by removing the ligature, when the effusion of lymph has completely obliterated the diseased veins, without waiting for it to cut entirely through the enclosed parts. But should it be deemed necessary in certain extreme cases to have this division effected, thereby to more completely prevent the disposition to return of the disease, as when the effusion of lymph did not seem sufficiently abundant, we can accomplish the result the more readily by this method, which gives us the power to tighten the loop from time to time, in proportion as it becomes loosened.

By keeping the cavity of the veins in the grasp of the ligature thus *constantly* closed, the risk of purulent absorption from the veins below is greatly diminished, if not entirely removed; for the constituents of the cord above the site of the operation are scarcely at all affected. The details of the operation are given for the left side, for it is upon that, almost exclusively, that the disease is found to exist, in consequence, it is most probable, of the entry of the left spermatic veins into the emulgent at a right angle to the course of the latter; while those of the right open into the vena cava nearly parallel with the course of that vessel.

Yours, very sincerely.
J. Pancoast.

*Philadelphia, Feb.* 28, 1843.

